# Patterns and correlates of old-age social exclusion in the Balkan states

**DOI:** 10.1007/s10433-023-00762-1

**Published:** 2023-05-04

**Authors:** Marja J. Aartsen, Marian Vasile, Laura A. Tufa, Diana A. Dumitrescu, Rosa M. Radogna, Jonathan Wörn, Iuliana Precupetu

**Affiliations:** 1 NOVA - Norwegian Social Research, Oslo Metropolitan University, Oslo, Norway; 2 University of Bucharest, Bucharest, Romania; 3 Romanian Academy, Bucharest, Romania; 4 Research Institute for Quality of Life, Bucharest, Romania; 5 Research Institute of the University of Bucharest, Bucharest, Romania; 6 Norwegian Institute of Public Health, Oslo, Norway

## Abstract

Social exclusion (SE), or the separation of individuals and groups from mainstream society, is associated with poor health and wellbeing, yet a substantial number of older people are socially excluded. There is increasing agreement that SE is multidimensional, comprising among others social relations, material resources, and/or civic participation. However, measuring SE is still challenging as exclusion may occur in more than one dimension, whereas its sum does not reflect the content of SE. To account for these challenges, this study provides a typology of SE and describes how SE types differ from each other in terms of severity and risk factors. We concentrate on Balkan states, which are among the European countries with the highest prevalence of SE. Data come from the European Quality of Life Survey (*N* = 3030, age 50 +). Latent Class Analysis revealed four SE types: low SE risk (50%), material exclusion (23%), material and social exclusion (4%), and multidimensional exclusion (23%). A higher number of dimensions from which a person is excluded are associated with more severe outcomes. Multinomial regression further revealed that lower levels of education, lower subjective health, and lower social trust increase the risks of any SE type. Younger age, unemployment, and not having a partner are associated with specific SE types. This study is in line with the limited evidence that different types of SE exist. Policies designed to reduce SE should take account of the different SE types and specific associated risk factors in order to enhance the impact of interventions to reduce social exclusion.

## Introduction

Social exclusion (SE)—or exclusion from mainstream society—is a significant societal problem as it threatens social cohesion, reduces an individual’s health and well-being, and increases loneliness (Dahlberg et al. 2022) and mortality (Dahlberg and McKee 2018; Lee 2021; Lennartsson et al. 2021; Sacker et al. 2017). The issue of SE is even more significant for older people (Dahlberg et al. 2020), as they have a higher risk of being socially excluded and to be excluded for a longer duration than younger age groups (Scharf and Keating 2012). Interventions to reduce SE have had a limited effect, partly because of inadequate scientific understanding of the different manifestations of SE (Byrne 2005; Walsh et al. 2017) and partly because of a narrow definition of SE, which leaves certain groups of excluded people undetected. For example, EU policies defined SE until recently mainly in terms of poverty, material deprivation, or living in a household with very low work intensity (European Commission 2010). However, scholars converge in their opinion that SE should be defined in broader terms to include, among other things, exclusion from social relations and exclusion from welfare state entitlements (Barry 1998; Burchardt et al. 1999; Walsh et al. 2017; Van Regenmortel et al. 2018), and the EU definition now also includes social deprivation (Eurostat 2021).

The number of studies on SE has grown steadily over the last few years, and while definitions depend on the scientific discipline and context (Walsh et al. 2017), SE is increasingly perceived as ‘… a complex and multi-dimensional process. It involves the lack or denial of resources, rights, goods and services, and the inability to participate in the normal relationships and activities, available to the majority of people in a society, whether in economic, social, cultural or political arenas. It affects both the quality of life of individuals and the equity and cohesion of society as a whole’ (Levitas et al. 2007). Based on this definition and on the international literature, Walsh and others (2017) developed an old-age exclusion framework consisting of six dimensions and many subdimensions, which are a key part of this study, namely material resources, social relations, services, civic and political participation, and neighbourhood and communities.

The multidimensionality of SE raises the question of whether different manifestations exist, or combinations of dimensions from which a person is excluded, and whether they differ in severity (Levitas et al. 2007). However, little is known about SE types, their risk factors, and severity. While it is assumed that ‘deep exclusion’ or the simultaneous exclusion from many domains may result in more ‘severe negative consequences for quality of life, well-being and future life-changes’ (Levitas et al. 2007, p. 9), there is not much evidence to support this claim. The aim of this study is therefore to (1) identify different types of SE among people living in countries with high risks of SE and (2) to evaluate associations between SE types, risk factors, and well-being outcomes.

This study focusses on the Balkans, a specific geographical area in the south-east of Europe consisting of states with similar histories of totalitarian regimes and poor welfare state entitlements. Not surprisingly, the Balkans are among the countries in Europe with the highest risks on many dimensions of SE (Sumil-Laanemaa et al. 2021; Morgan et al. 2021) and between 20 and 48% of the people aged 60 + living in Balkan states is socially excluded according to EU definitions (Eurostat 2021). This study further focusses on the individual aspects of SE, since variations in macro-level variables are most likely limited.

## Literature

A fundamental issue in debates about SE is whether exclusion is associated with individuals’ specific preconditions (risk factors) or whether it is the social structure that excludes people from mainstream society (drivers). Another factor that is sometimes discussed is the degree to which exclusion is a voluntary act of the individual (self-exclusion). However, while people say they choose for SE to escape from social pressures or to relax (Lay et al. 2020), it may be difficult to determine whether it is truly voluntary (Barry 1998; Victor et al. 2008; Weldrick and Grenier 2018, Lay et al. 2020).

Literature on risk factors of SE indicates that age, gender, education, household size, and partner status are among the most frequently observed (e.g. Burholt and Aartsen 2021; Sumil-Laanemaa et al. 2021; Myck et al. 2021). Educational attainment and migrant status are related to multiple forms of exclusion (Scharf et al. 2005). The level of material resources in one generation affects material exclusion in the next generation via intergenerational transmission of (dis)advantages (Peruzzi 2015). Transitions during the life course, such as from employee to retiree, and events, such as the loss of a spouse, can lead to exclusion from social and material resources (Dewilde 2003). Health and functioning are important conditions for various forms of, for example, civic and social participation (Sacker et al. 2017), and lack of trust as requirement for social engagement, can lead to social disengagement (Rapolienė and Aartsen 2021). Important factors at the meso-level are a lack of a sense of belonging to a neighbourhood, and living in a deprived area, both of which are related to increased levels of exclusion (Prattley et al. 2020; Dahlberg and McKee 2018). Gender is a cross-cutting factor which may moderate the many potential associations between risk factors and SE (Aartsen et al. 2021), potentially leading to a disproportionately higher chance that older females become socially excluded (Dahlberg et al. 2020).

Recent findings suggest that SE is a dynamic construct as people can move in and out states of SE, although there is a tendency for exclusion to increase over the course of life as exclusion from one dimension increases the risk of exclusion from other dimensions (Scharf et al. 2021; Dahlberg 2021). Evidence further suggests that the dimensions of exclusion are connected (Scharf et al. 2021; Dahlberg 2021). Exclusion from one dimension increases the risk of exclusion from other dimensions. People are not necessarily excluded from all dimensions simultaneously. And while in theory a myriad of combinations of dimensions can exist, certain combinations are more prevalent than others, whereas other combinations hardly occur. For example, in the empirical studies in Belgium (Van Regenmortel et al. 2018) and the UK (Becker and Boreham 2009) only a limited number of combinations, or types of SE, are observed.

In line with previous empirical research, we thus anticipate a limited number of SE types. Risk factors are selected based on their expected relation with SE type, and we expect that older age, female gender, a low degree of urbanisation, no partner, low subjective health, a low level of education, migrant status, no employment, and low trust in other people and in the parliament are associated with an increased risk of any SE type. In line with life course theories on the cumulation of (dis)advantages (e.g. Ferraro et al. 2009), we expect a linear association between the number of dimensions from which a person is excluded, and the severity of SE. Severity will be expressed in terms of loneliness and mental well-being, as loneliness and well-being are associated with all domains of SE (Dahlberg et al. 2022; Precupetu et al. 2019).

## Methods

### Research design

This cross-sectional study is based on data from the European Quality of Life Survey (EQLS) from 2016, which was, at the time of the study, the most recent data collected (Eurofound 2018). EQLS is a repeated cross-sectional survey of people aged 18 + living in private households. The objective is to improve the living and working conditions of European citizens. The survey examines both the objective circumstances of people’s lives, such as employment, income, education, housing, family, and health, as well as subjective questions about, for example, life satisfaction and the perceived quality of the society in which people live. Data were collected by means of computer-assisted face-to-face interviews at respondents’ homes. The interviewers adhered to a set of rules governing their conduct and the confidentiality of the project. Survey participation was based on voluntary informed consent that was verbally obtained (Eurofound 2018).

Our study sample (*N* = 3030) consists of older people living in Balkan states, including Bulgaria (20% of the study sample), Romania (18%), Albania (17%), Montenegro (11%), Macedonia (17%), and Serbia (18%). We selected people aged 50 + to retain sufficient power and to acknowledge that socially excluded individuals have a higher risk of mortality (Holt-Lunstad et al. 2010). The average age of our study sample is 64 (SD = 9.3). Slightly more than half of the sample (56%) are female.

#### Measurements

The operationalisation of SE is based on the conceptual framework by Walsh and others (2017) who distinguish six different domains of exclusion, i.e. social relations, material and financial resources, services amenities and mobilities, civic participation, neighbourhood and community, and socio-cultural aspects. Several subdimensions are defined for each domain, and these are used to guide the selection of variables in our analytical models. All domains are included, except for socio-cultural aspects. These aspects refer to macro-social characteristics of a society which cannot be examined in a collection of countries with similar macro-social contexts. Furthermore, the previous practices in the measurement of old-age SE are adhered to, in which multiple indicators per dimension are used and in which the multidimensionality is preserved (Levitas et al. 2007; Kneale 2012; Van Regenmortel et al. 2018).

##### SE domains and subdimensions

The domains included in our SE variable are (1) civic participation, (2) services, (3) financial resources, (4) neighbourhood, and (5) social relations, and for each domain three subdimensions were selected (Table 1). To avoid very sparse tables and in accordance with general practice (Nylund-Gibson and Choi 2018), all indicators are dichotomised into 1 (indicative for inclusion) and 0 (indicative for exclusion). In this study, *civic participation* includes (1) participation in church and/or social clubs at least once a month (1 = yes/0 = no), (2) feeling left out of society (neutral or (strongly) disagree = 1/(strongly) agree = 0), and (3) participation in at least one of the six following political activities in the last year: attended a meeting of a trade union, a political party or political action group; attended a protest or demonstration; signed a petition, including an e-mail or online petition; contacted a politician or public official; commented on a political or social issue online; boycotted certain products (1 = at least one activity/0 = else). Exclusion from *services* includes (1) can afford general practitioner (GP), family doctor or health-care services (1 = yes/0 = only with (great) difficulty); (2) have been online in the last year (1 = yes/0 = no); and (3) satisfaction with the GP. For satisfaction with the GP, a scale was developed based on the following four items: quality of the facilities, expertise and professionalism of staff, personal attention, and being informed or consulted about health care. Response categories ranged from 1 to 10 with higher scores indicating higher satisfaction (Cr. alpha = 0.92). The scale was dichotomised using an arbitrary cut-off of 7 into satisfied (1 = 7 or higher) and not satisfied (0 = lower than 7). For exclusion from *material resources*, the following categories were selected (1) being able to make ends meet (1 = yes/0 = only with (great) difficulty); (2) being able to keep the home adequately warm (1 = yes/0 = no); and (3) material deprivation. Material deprivation is measured by asking whether the respondent is able to afford the following items: paying for a week's annual holiday, replacing worn-out furniture, affording a meal with meat/chicken/fish every second day if desired, buying new rather than second-hand clothes, having friends or family for a drink or meal once a month (1 = four or five items can be afforded/0 = three or fewer items can be afforded). For *neighbourhood exclusion, the following categories* were selected (1) feeling safe walking alone after dark (1 = yes/0 = no); (2) feeling close to people in the area where they life (1 = (strongly) agree/0 = neither agree or disagree and (strongly) disagree); and (3) access to banking facilities, transport, culture, green area, and grocery stores. Scores relating to access to facilities reflect the average score on each item, which are dichotomised into 1 (rather or very easy) and 0 (rather or very difficult). Exclusion from *social relations* includes (1) having contact with family and relatives at least once a month (1 = yes/0 = no); (2) having contact with neighbours and friends at least once a month (1 = yes/0 = no); and (3) satisfaction with family life. Response categories for satisfaction with family life ranged from 1 to 10 with higher scores indicating higher satisfaction. The scale was dichotomised using an arbitrary cut-off of 7 into satisfied (1 = 7 or higher) and not satisfied (0 = lower than 7).

**Table 1:** Initial selection of SE dimensions and subdimensions and thresholds for exclusion

Dimension	Subdimension	Indicative of exclusion
Civic participation	Participation in church and/or social clubs at least once a month	No
	Feeling left out of society	(Strongly) agree
	Participation in political activities in the last year	No
Services	Can afford to see a general practitioner (GP), family doctor or health-care services	Only with (great) difficulty
	Having been online in the last year	No
	Satisfaction with the GP	Scale score 6 or lower out of 10 (max)
Financial resources	Being able to make ends meet	Only with (great) difficulty
	Being able to keep the home adequately warm	No
	Material deprivation*	At least 3 out of 5 items cannot be afforded
Neighbourhood	Feeling safe when walking alone after dark	No
	Feeling close to people in the area where they life	Neither agree nor (strongly) disagree
	Access to banking facilities, transport, culture, green area, and grocery stores	Rather or very difficult
Social relations	Contact with family and relatives at least once a month	No
	Contact with neighbours and friends at least once a month	No
	Satisfied with family life	Scale score 6 or lower out of 10 (max)

*Material deprivation is measured by asking whether the respondent is able to afford the following items: paying for a week's annual holiday, replacing worn-out furniture, affording a meal with meat/chicken/fish every second day if desired, buying new rather than second-hand clothes, having friends or family for a drink or meal once a month

##### Risk factors of SE

We also selected a wide range of potentially relevant risk factors of SE, which have been observed in previous studies and were available in the data: degree of urbanisation, partner status, subjective health, level of education, migrant status, employment status, trust in people and trust in parliament, age, and gender. *Urbanisation* is a categorical variable describing the area in which people live, with the following categories (1) the open countryside, (2) a village/small town, (3) medium to large town, (4) city or city suburb. *Partner status* is based on legal marital status, which is dichotomised into 0 = no partner (including the categories widowed, separated, divorced, and never married) and 1 (married). *Subjective health* is based on the question ‘In general, how is your health?’ and has 5 response categories. The response categories were recoded so that higher scores indicate better subjective health. *Level of education* is based on the International Standard Classification of Education (ISCED) and has nine categories, ranging from 0 (low education) to 8 (high education). *Migrant status* is a dichotomous variable indicating whether a person is born in another country than the surveyed country (1 = yes/0 = no). *Employment status* is based on a 12-category variable, which are recoded into employed (1 = employed/employed but on paid leave) and unemployed (0 = retired, unemployed, no paid work, unable to work due to long-term illnesses). *Trust in people* is assessed by asking people whether they agree with the statement ‘Do you trust other people?’, with response categories ranging from 1 (you cannot be too careful) to 10 (most people can be trusted). *Trust in the parliament* is assessed by asking people whether they agree with the statement ‘Do you trust the parliament in your country?’ with response categories ranging from 1 (do not trust at all) to 10 (trust completely). Age reflects the number of years lived and gender is a dichotomous variable with (1) for male and (2) for female.

##### Outcomes of SE

Loneliness was measured with a single-item statement ‘Over the last two weeks I felt lonely’ with six response categories recoded so that higher scores indicate greater loneliness. Mental well-being was assessed using the World Health Organization Wellbeing Index (WHO-5 scale) which has adequate validity (Topp et al. 2015). The WHO-5 scale consists of the following five questions: (1) I have felt cheerful and in good spirits, (2) I have felt calm and relaxed, (3) I have felt active and vigorous, (4) I woke up feeling fresh and rested, and (5) my daily life has been filled with things that interest me. Answers refer to the last 14 days and response categories ranged from 1 (all the time) to 6 (none of the time). The internal consistency for these five items was also high in our sample (Cr. alpha = 0.93). The response categories have been recoded so that higher scores indicate higher well-being, and an average score has been calculated.

#### Analytical strategy

The study examines whether different manifestations or types of SE exist, whether these types differ in severity, and how they are associated with potential risk factors. Analyses were conducted in three subsequent steps. In the *first step,* SE types were identified by means of Latent Class Analysis (LCA). LCA is a statistical clustering technique that organises the whole sample of heterogeneous people into smaller, more homogenous subgroups of people (Hagenaars and McCutcheon, 2002) with similar manifestations of social exclusion. Each subgroup represents a different SE type. The decision on the number of SE types with LCA was based on a combination of formal statistics and on the theoretical meaningfulness of the different SE types. The formal statistical tests include fit indices (smallest Bayesian information criterium (BIC), and smallest adjusted BIC (adj. BIC)), high classification quality (that is an entropy close to 1), and parsimony (based on the Vuong-Lo-Mendell-Rubin Likelihood Ratio test for *K*-1 versus *K* classes; Jung and Wickrama 2008). These tests make LCA superior to the more traditional cluster techniques such as *K*-means, which do not provide such statistics (Magidson and Vermunt 2002). The second criterium, that is the theoretical meaningfulness of the different subtypes, has decisive importance if the various formal statistics suggest different numbers of SE types. Two parameters are important to decide on the meaningfulness of SE types, i.e. the conditional probability and the latent class probability. The conditional probability is the probability that a person with a specific SE type will be at a specific level of the various dimensions, e.g. will have social resources, or material resources, or any other dimension of SE, given the SE type to which he or she belongs. Latent class probabilities reflect the prevalence of each SE type. For reasons of parsimony, dimensions with similar conditional probabilities across the SE types are excluded, as these dimensions discriminate poorly between the SE types. For each person in the sample, the most likely SE type was identified, saved, and merged with the datafile for the analyses in steps 2 and 3. The LCA was conducted with MPlus version 8.4 (Muthén and Muthén 1998–2017).

The *second step* evaluates whether the average level of mental well-being and loneliness differed by SE type, by means of an ANOVA, and in the *third step* we examined multivariate associations between the potential risk factors of SE by means of a multinomial regression with SE types as dependent variable and low SE risk as reference group. Urbanisation and subjective health were treated as categorical variables with more than two categories in the model, and gender, partner status, migrant status, and employment as dichotomous variables.

## Results

The basic characteristics of the study sample are presented in Table 2. The proportion of males is below the EU average in all countries, except Montenegro, which may reflect the relatively large gender gap in life expectancy, with women living longer than men in many of the Balkan states (https://stat.link/042196). Compared to the other EU countries, people in Balkan states have lower education (except in Bulgaria), lower employment rates (except in Montenegro), are less often migrants (except in Serbia), have lower trust in other people or the parliament, and slightly lower subjective health (except in Bulgaria). The prevalence of living with a partner is higher in all Balkan states, except in Romania, where it is closer to the EU average. Overall, people in Balkan states are, or feel, more often excluded than in other EU countries in almost all dimensions, except contact with family (more often contact in Albania, Montenegro, Macedonia, and Serbia), contact with neighbours and friends (more often contact in Bulgaria, Albania, Montenegro, and Serbia), and, in some of the Balkan states, more people feel safe walking alone after dark (Montenegro, Macedonia, and Serbia) than in the other EU countries.

**Table 2:** Descriptive characteristics of study sample by Balkan states and EU* average

	Bulg.	Rom.	Alb.	Monten.	Maced.	Serbia.	EU*
Mean Age (range 50–95)	65.4	66.1	63.0	60.2	64.3	62.6	65.3
Gender (% Male)	41.1	35.1	43.9	52.9	46.3	47.9	56.9
Mean level of education (range 0–8)	3.5	2.6	2.9	3.1	2.5	1.4	3.2
Partner status (% with partner)	56.1	54.4	79.9	61.7	68.8	65.3	55.6
Being employed (%)	29.2	15.5	16.8	30.6	22.8	29.3	29.8
*Urbanisation*							
Open countryside (%)	0.0	0.2	6.2	2.9	3.4	1.6	12.5
Village/small town (%)	52.2	58.6	43.9	43.9	39.7	46.1	42.5
Medium to large town (%)	16.0	23.0	9.4	43.6	31.3	40.1	24.4
City or city suburb (%)	31.8	18.2	40.5	9.5	25.5	12.2	20.5
Migrant status (%)	1.0	0.5	0.6	9.0	3.3	12.2	8.8
Mean subjective health (0–5, 5 = high)	3.4	2.9	3.2	3.3	3.3	3.2	3.4
Mean trust in other people (1–10, 10 = high)	3.9	4.8	2.6	4.2	2.9	4.2	5.0
Mean trust in the parliament (1–10, 10 = high)	3.4	4.0	4.3	3.2	3.9	5.3	4.7
*Indicators of the SE types (% yes)*							
Participation in social and religious organisations	17.9	42.0	24.7	37.0	36.4	41.6	50.7
Feeling included in society	57.6	70.8	78.2	64.5	62.6	55.0	78.0
Participation in political activities	14.2	7.2	14.2	17.6	15.1	19.4	26.8
Can afford health-care facilities	79.7	57.3	65.2	54.1	89.4	68.2	80.4
Being online in the last year	5.8	3.9	8.1	12.0	11.1	11.5	10.6^**^
Satisfied with GP	70.7	77.0	76.1	51.1	70.0	58.2	81.2
Being able to make ends meet	58.8	53.7	41.6	72.1	75.0	61.3	82.5
Being able to keep the house warm	73.9	75.8	49.0	83.3	84.7	83.5	89.5
Being able to afford at least four out of five items	45.3	39.1	27.4	50.8	42.9	53.0	76.8
Feeling safe walking alone after dark	72.7	79.6	81.6	86.9	90.6	88.7	83.8
Feeling close to the people in the area	78.2	68.6	87.1	59.4	77.4	55.7	71.2
Easy access to facilities	67.1	52.2	58.3	63.5	66.5	56.7	69.5
Contact with family and relatives at least once per month	84.8	77.7	88.1	96.5	89.4	92.5	88.8
Contact with neighbours and friends at least once per month	94.8	88.2	93.9	97.7	88.7	97.1	93.1
Satisfied with family life	57.0	67.5	77.0	63.5	66.9	70.4	77.0
N	593	556	513	346	523	499	16.265

Bulg. = Bulgaria, Rom. = Romania, Alb. = Albania, Monten. = Montenegro, Maced. = FYR of Macedonia,

*GP*, General practitioner

*EU Average of all other countries in the EQLS that are part of the EU, **52.8% missing observations

The results of the latent class analysis are presented in Table 3. As is often the case with LCA, the formal fit statistics were not unequivocal about the number of SE types. As can be seen in Table 3, the (adjusted) BIC indicates that the best model includes five types of SE (smallest BIC), whereas the accuracy of classifying people in the right SE type is highest when a two-SE-type solution is chosen. The most parsimonious model distinguishes three SE types. The decisive factor for determining the number of SE types was the solution that gave the most nuanced description of SE, while at the same time preserving parsimony. This was the model with four SE types. The conditional probabilities and latent class sizes are given in Table 4. Table 4 can be interpreted as follows: the third row of the full model suggests that people in type I have a high probability of feeling included in society (*p* = 0.81), whereas people with SE type II have a low probability of feeling included in society (*p* = 0.26). For people with SE type III and IV the proportions are round 0.50, indicating an equal proportion of people who feel included in society as people who do not feel included. From the second row of the full model, it follows that the probability of participating in a social or religious organisation is rather similar for the four SE types. A similar reasoning can be followed for the indicators participation in political activities, being online in the last year, and feeling safe when walking alone after dark. To further optimise the model fit, all indicators that did not discriminate between the four SE types were removed from the 4-class solution. This trimmed model had a lower (adjusted) BIC and higher entropy (last row Table 3).

**Table 3:** Fit statistics and proportions of SE types based on the most likely class membership

# Class	BIC	Adj. BIC	L-ratio for *K*-1 versus *K*	Entropy	Proportions for the SE types				
					I	II	III	IV	V
2	47,590.49	47,491.99	0.00	0.71	0.52	0.48			
3	47,477.35	47,328.01	0.00	0.65	0.52	0.24	0.24		
4	47,422.84	47,222.66	0.68	0.69	0.51	0.22	0.18	0.09	
5	47,394.96	47,143.94	0.23	0.62	0.34	0.24	0.21	0.17	0.04
Trimmed model	34,346.13	34,196.79		0.73	0.50	0.23	0.04	0.23

**Table 4:** Proportions SE types based on most likely class membership and conditional probabilities (*N* = 3030)

		Full model				Trimmed model			
		I	II	III	IV	I	II	III	IV
Proportions based on most likely class membership	Total sample	0.51	0.22	0.18	0.09	0.50	0.23	0.04	0.23
Civic participation (% yes)									
Participation in social or religious organisations	(0.32)	0.35	0.40	0.28	0.23				
Feeling included in society	(0.64)	0.81	0.26	0.43	0.69	0.81	0.26	0.60	0.72
Participation in political activities	(0.14)	0.18	0.15	0.07	0.09				
Access to services (% yes)									
Can afford health-care facilities	(0.69)	0.84	0.54	0.49	0.57	0.84	0.56	0.59	0.57
Being online in the last year	(0.07)	0.09	0.08	0.04	0.04				
Satisfied with GP	(0.46)	0.47	0.26	0.43	0.61	0.74	0.43	0.69	0.84
Material resources (% yes)									
Being able to make ends meet	(0.59)	0.92	0.37	0.29	0.18	0.93	0.31	0.45	0.23
Being able to keep the house warm	(0.74)	0.96	0.54	0.50	0.54	0.96	0.55	0.59	0.51
Being able to afford at least four out of five items	(0.40)	0.73	0.10	0.11	0.05	0.78	0.11	0.21	0.04
Neighbourhood (% yes)									
Feeling safe walking alone after dark	(0.82)	0.89	0.76	0.68	0.78				
Feeling close to the people in the area	(0.71)	0.75	0.45	0.55	0.94	0.75	0.48	0.58	0.91
Easy access to facilities	(0.60)	0.72	0.46	0.44	0.55	0.72	0.45	0.55	0.55
Social resources (% yes)									
Contact with family and relatives at least once per month	(0.86)	0.91	1.00	0.23	0.94	0.92	0.87	0.00	0.90
Contact with neighbours and friends at least once per month	(0.93)	0.95	0.99	0.59	0.96	0.97	0.96	0.00	0.95
Satisfied with family life	(0.66)	0.81	0.36	0.40	0.71	0.82	0.32	0.41	0.75

SE types I = Low SE risk, II = Material exclusion, III = Exclusion from material resources and social relations, IV = Multidimensional exclusion, GP = General Practitioner

The latent class probabilities (first row Table 4) indicate for the trimmed model that the prevalence of people with SE type I, II, III, and IV is 50, 23, 4, and 23 per cent, respectively. Based on the conditional probabilities, Type I is labelled as ‘*Low SE risk*’, as the probability of exclusion was low for all indicators. Type II is characterised by exclusion from material resources, but not exclusion from other dimensions. Type II is labelled ‘*Material exclusion*’. Type III was mainly characterised by having no contact with family, relatives, neighbours, and friends, and a low quality of family life. The probability that people can afford to buy new items as well as being able to make ends meet was also low. Type III is therefore labelled ‘*Exclusion from material resources and social relations*’. Type IV is characterised by low feelings of societal inclusion, low satisfaction with the GP, low levels of material resources, disconnection from neighbours, and low access to services. In addition, while people in this type are not excluded from social relations, the satisfaction with family life is low. Type IV is therefore labelled ‘*Multidimensional exclusion*’.

By means of a subsequent ANOVA, we observed significant differences in severity of SE pertaining to loneliness across the four SE types (*F* = 203.12, df = 3, *p* < 0.01). The lowest levels of loneliness were observed in people with low SE risk (*M* = 1.97, 95% CI = 1.91–2.04), followed by people excluded from material resources (*M* = 2.46, 95% CI = 2.34–2.59), people excluded from material and social resources (*M* = 3.40, 95% CI = 3.05–3.75), and the highest average level of loneliness was observed among people with multidimensional exclusion (*M* = 3.58, 95% CI = 3.46–3.69). Significant differences were also observed in the severity of SE pertaining to mental well-being across the four groups (*F* = 164.30, df = 3, *p* < 0.01), with highest levels of well-being observed in people with low SE risk (*M* = 4.21, 95% CI = 4.16–4.26), followed by people excluded from material resources (*M* = 3.60, 95% CI = 3.50–3.69), and people excluded from material resources and social relations (*M* = 3.25, 95%CI = 3.00–3.51). People excluded from multiple dimensions have the lowest mental well-being (*M* = 3.11, 95% CI = 3.03–3.20) (results not in a table).

**Fig. 1:**
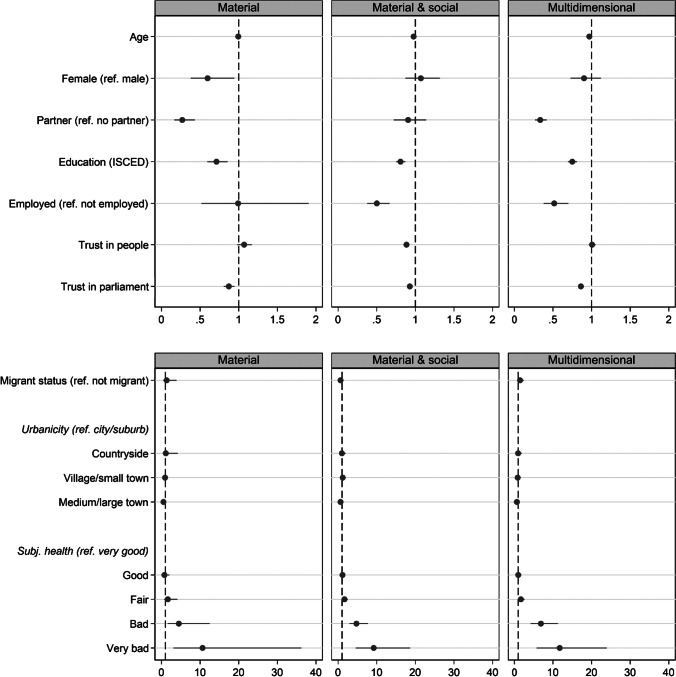
Plot of the parameter estimates and 95% confidence intervals (CI) for the multinomial regression of SE type on the study variables (*N* = 3030). Note that the *x*-axis of the upper two figures is different from the lower two figures due to the large CI’s for the small groups

Finally, we estimated a multinomial model where potential risk factors of SE were regressed on SE types, with low SE risk as reference category. Several risk factors appeared to be significantly associated with some or all SE types (Fig. 1). Low education, low trust in parliament, living in a medium to large town as compared to living in the city, and lower subjective health increase the odds of being excluded, irrespective of the type of SE. Being younger, unemployed, and having low trust in other people is additionally associated with an increased risk of exclusion from material resources; being male and living without a partner is additionally associated with a higher risk from material and social resources, and being younger, unemployed, and living without a partner is additionally associated with a lower risk of multidimensional exclusion.

## Discussion

This study provides evidence of the existence of different types of SE and indicates various factors that are associated with SE in older people living in countries with high SE prevalence. An estimation has been made of the prevalence of each SE type, and a broad range of risk factors and outcomes of SE have been investigated. The study finds that half of the people aged 50 + in Balkan states are not excluded from mainstream society, which also means that half of the 50 + population in the Balkan states are excluded from one or more dimensions of SE. There is evidence for four types of SE: low risk of exclusion (observed for 50% of the 50 + people in our sample), exclusion from material resources (23%), exclusion from material resources and social relations (4%), and exclusion from material resources, neighbours, and society, with low satisfaction with the family and general practitioner, here referred to as multidimensional exclusion (23%). All our proposed risk factors, except migrant status, have been found to be associated with some or all SE types, although not all in the expected direction. Low education, low trust in the parliament, living in a medium to large town as compared to living in the city, and lower subjective health are associated with all types of SE; being unemployed is only associated with material and multidimensional exclusion; living without a partner is only associated with exclusion from material and social resources, and multidimensional exclusion; and lower trust in other people is only associated with exclusion from material resources.

In contrast to our expectations, it was observed that, compared to older-old people, younger-old people have an increased risk of exclusion from material resources and multidimensional exclusion. One explanation for this is that older people may own a mortgage-free property and do not need to pay rent, making it easier to make ends meet (Age Platform Europe 2018). An alternative explanation for this may be that it is not age per se but being unemployed which increases the risk for exclusion from material resources (Matkovic 2006). It is unclear whether it is the exclusion from material resources that drives the significant association between multidimensional exclusion and age. This would need further investigation.

Also in contrast with our expectation is that it is men, and not women, that have an increased risk of material and social exclusion, although no gender differences for material exclusion or multidimensional exclusion are observed, which is in line with studies in the UK (Scharf et al. 2005) and Australia (Miranti and Yu 2015). It is unclear why men in Balkan states are at increased risk of material and social exclusion, and any explanation would be speculative. It may be, for example, that prevailing gender norms surrounding social roles leave men more vulnerable to SE than women. The male breadwinner norm is still dominant in Balkan countries, and those who cannot conform to expectations likely withdraw from social relations and/or are stigmatised, leading to a common experience of being excluded from both material resources and social connections.

In line with previous claims and findings that SE leads to lower well-being (Walsh et al. 2017; Sacker et al. 2017), this study finds that the higher the number of domains from which a person is excluded, the higher their loneliness and the lower their well-being. Hence, exclusion from multiple domains is the most severe form of SE. As can also be seen in the sparse number of empirical studies, while many manifestations of SE may exist, only a limited number are observed, even in countries with high SE risks. It is apparent that domains of exclusion tend to cluster around three types of SE: material exclusion, social and material exclusion, and exclusion from many domains simultaneously, or multidimensional exclusion. It was also observed that factors associated with SE are partly specific, only affecting one or two types of SE, and partly generic, affecting all types of SE, suggesting qualitative differences in the (origins of) SE.

There are limitations to this study. Firstly, the selection of SE indicators is confined to variables available in the data used, meaning that the most ideal indicators could not always be selected. Secondly, cross-sectional data were used, which makes it impossible to separate drivers, risk factors, indicators, and outcomes of SE (Macleod et al. 2019). However, distinguishing between the various associated factors is not so relevant to the purpose of this study. Thirdly, data were combined from six countries. Although all those countries had a relatively high degree of SE—similar welfare regimes, and comparable histories in respect of the many transformations in economic structures, labour markets, and political institutions (Precupetu et al. 2019)—variations and different patterns of SE may still exist. A hierarchical or multilevel LCA can take this regional or country-level variation into account (Pirani 2013). However, since the study only has 6 level-2 units, and because 50 or more level-2 units are recommended for an accurate estimation of standard errors (Maas and Hox 2005), a multilevel LCA was not conducted.

Despite its limitations, this study has contributed to a deeper understanding of SE in several important ways. Firstly, while a large variation of different manifestations of SE might be expected, evidence suggests that the number of SE types is limited, even in countries with a high SE risk. Thinking in types of SE helps to reduce the inherent complexity of a multidimensional concept, which makes it easier to develop strategies to reduce SE. Secondly, there appears to be generic and specific risk factors of the SE types, and SE types differ in severity, which makes each SE type qualitatively distinct. Thirdly, SE types differ in terms of severity, with the number of exclusion dimensions linearly related to higher levels of loneliness and lower levels of mental well-being.

The results of this study emphasise the importance of tailoring policy interventions to the various types or manifestations of SE, while also considering the different risk factors. Policies should continue to address the high levels of material exclusion in Balkan states and in other poorer countries. The range of income-centric policies (e.g. guaranteed minimum income, benefits for those suffering from fuel poverty, disability benefits) could be expanded and better targeted to those in need. In addition to income maintenance policies for older people, preventive strategies should also concentrate on those currently active in the labour market who have a greater risk of low income after retirement. This includes, for example, people working in the grey economy, persons with fragmented work trajectories, and those who experience long-term unemployment. Exclusion from material resources and social relations, as well as multidimensional exclusion, highlights the importance of implementing the principles of active ageing, especially when creating socially supportive environments for older people. Programmes to sustain extended social relationships in the community, such as intergenerational interaction programmes, supporting communities or clubs that centre on specific activities that bring people together, could be particularly beneficial. Such programmes could expand spheres of sociability beyond close relationships and help secure a social network that withstands disruptive life events such as the death of a partner, separation, or divorce. A broader social sphere may lessen the exclusive reliance on close family members that is typical in familistic welfare states.

In conclusion, while opinions increasingly converge about what SE encompasses and its different manifestations, more needs to be done. Firstly, the indicators for different dimensions vary across studies, which compromises the comparability of SE across countries and welfare states. Having said that, since SE is a relative concept, defining who is included or excluded depends on what is normal in a particular society, which implies that the threshold of each indicator can only be defined at a societal level. Secondly, many studies are cross-sectional which makes it impossible to separate risk factors, indicators, and outcomes of SE. There needs to be more research into macro-level drivers, such as state benefits for compensating exclusion from material resources or health-care services, and into cultural, gendered, or ageistic norms about older peoples. That requires longitudinal data and cross-national comparisons, and an analytical method that can handle these data. Hierarchical longitudinal LCA may be a good candidate for these types of analyses.

## Appendix

(See Table

**Table 5:** Parameter estimates for the multinomial regression of SE type on the study variables (*N* = 3030)

	Exp(B)	95% CI		Exp(B)	95% CI		Exp(B)	95% CI	
		Lower	Upper		Lower	Upper		Lower	Upper
	Material versus Low-risk SE			Material and social versus Low-risk SE			Multidimensional versus Low-risk SE		
Age	***0.98***	***0.97***	***0.99***	0.99	0.97	1.02	**0.97**	**0.96**	**0.98**
Female gender	1.07	0.87	1.32	***0.60***	***0.38***	***0.94***	0.90	0.73	1.12
With partner	0.91	0.72	1.14	***0.27***	***0.17***	***0.43***	***0.34***	***0.27***	***0.42***
Education	***0.81***	***0.75***	***0.87***	***0.71***	***0.59***	***0.85***	***0.75***	***0.69***	***0.81***
Migrant status	0.62	0.34	1.14	1.34	0.46	3.91	1.48	0.91	2.40
Being employed	***0.50***	***0.38***	***0.67***	0.99	0.52	1.91	***0.52***	***0.38***	***0.70***
Trust in people	***0.89***	***0.85***	***0.92***	1.07	0.98	1.17	1.01	0.96	1.05
Trust in the parliament	***0.93***	***0.90***	***0.96***	***0.87***	***0.81***	***0.94***	***0.86***	***0.83***	***0.90***
*Urbanity (ref is city or suburb)*									
Open countryside (%)	0.97	0.49	1.93	1.12	0.30	4.19	0.99	0.50	1.93
Village/small town	1.19	0.92	1.53	0.93	0.54	1.61	0.87	0.67	1.13
Medium to large town	***0.62***	***0.46***	***0.83***	***0.52***	***0.27***	***1.00***	***0.66***	***0.49***	***0.89***
*Subjective health (Ref* = *Very good)*									
Good	1.14	0.74	1.75	0.77	0.29	2.06	1.02	0.65	1.60
Fair	***1.64***	***1.07***	***2.51***	1.64	0.65	4.14	***1.69***	***1.09***	***2.62***
Bad	***4.74***	***2.92***	***7.70***	***4.48***	***1.61***	***12.47***	***6.88***	***4.19***	***11.28***
Very bad	***9.22***	***4.55***	***18.67***	***10.64***	***3.13***	***36.20***	***11.74***	***5.76***	***23.93***

Bold and italic numbers indicate that *p* < 0.05

^5^).
